# Supplementary material for: The chaperonin CCT8 controls proteostasis essential for T cell maturation, selection, and function
Source: Commun Biol. 2021 Jun 3;4:681. doi: 10.1038/s42003-021-02203-0 (PMC8175432; doi:10.1038/s42003-021-02203-0)
Supplement: Supplementary file 2 — Supplementary information. [file 42003_2021_2203_MOESM2_ESM.pdf]

Supplementary material

The chaperonin CCT8 controls proteostasis essential for T cell maturation, selection, and function

Bergithe E Oftedal, Stefano Maio, Adam Handel, Madeleine PJ White, Duncan Howie,
Simon Davis, Nicolas Prevot, Ioanna A. Rota, Mary E Deadman, Benedikt M Kessler, Roman Fischer,
Nikolaus S Trede, Erdinc Sezgin, Rick M Maizels, Georg A Holländer

Table of Content

Table S1. Statistical significance for the cell survival at different timepoints .... Page 2

Figure S1. Selection and maturation of cortical and medullary thymocytes in the absence of CCT8 expression .... Page 3

Figure S2. Proteomic analysis of activated CD4 cells .... Page 4

Figure S3. Peripheral T cell function in the absence of CCT8 expression .... Page 5

Figure S4. The response to *H. polygyrus* infection in CCT8-deficinent mice .... Page 6

Supplementary table 1. Statistical significance for the cell survival at different timepoints

**Time Point**	**Without IL-2**	**P-value**	
0 hrs	$CCT8^{T+/+}$ versus $CCT8^{T-/-}$	0,999546	ns
8 hrs	$CCT8^{T+/+}$ versus $CCT8^{T-/-}$	0,221398	ns
20 hrs	$CCT8^{T+/+}$ versus $CCT8^{T-/-}$	0,00271	**
96 hrs	$CCT8^{T+/+}$ versus $CCT8^{T-/-}$	0,018598	*
	**With IL-2**		
0 hrs	$CCT8^{T+/+}$ versus $CCT8^{T-/-}$	0,999995	ns
8 hrs	$CCT8^{T+/+}$ versus $CCT8^{T-/-}$	0,753643	ns
20 hrs	$CCT8^{T+/+}$ versus $CCT8^{T-/-}$	0,038117	*
96 hrs	$CCT8^{T+/+}$ versus $CCT8^{T-/-}$	0,001376	**

ns: not significant; * p<0.05; ** p<0.01

Supplementary Figure 1, related to main Figure 1

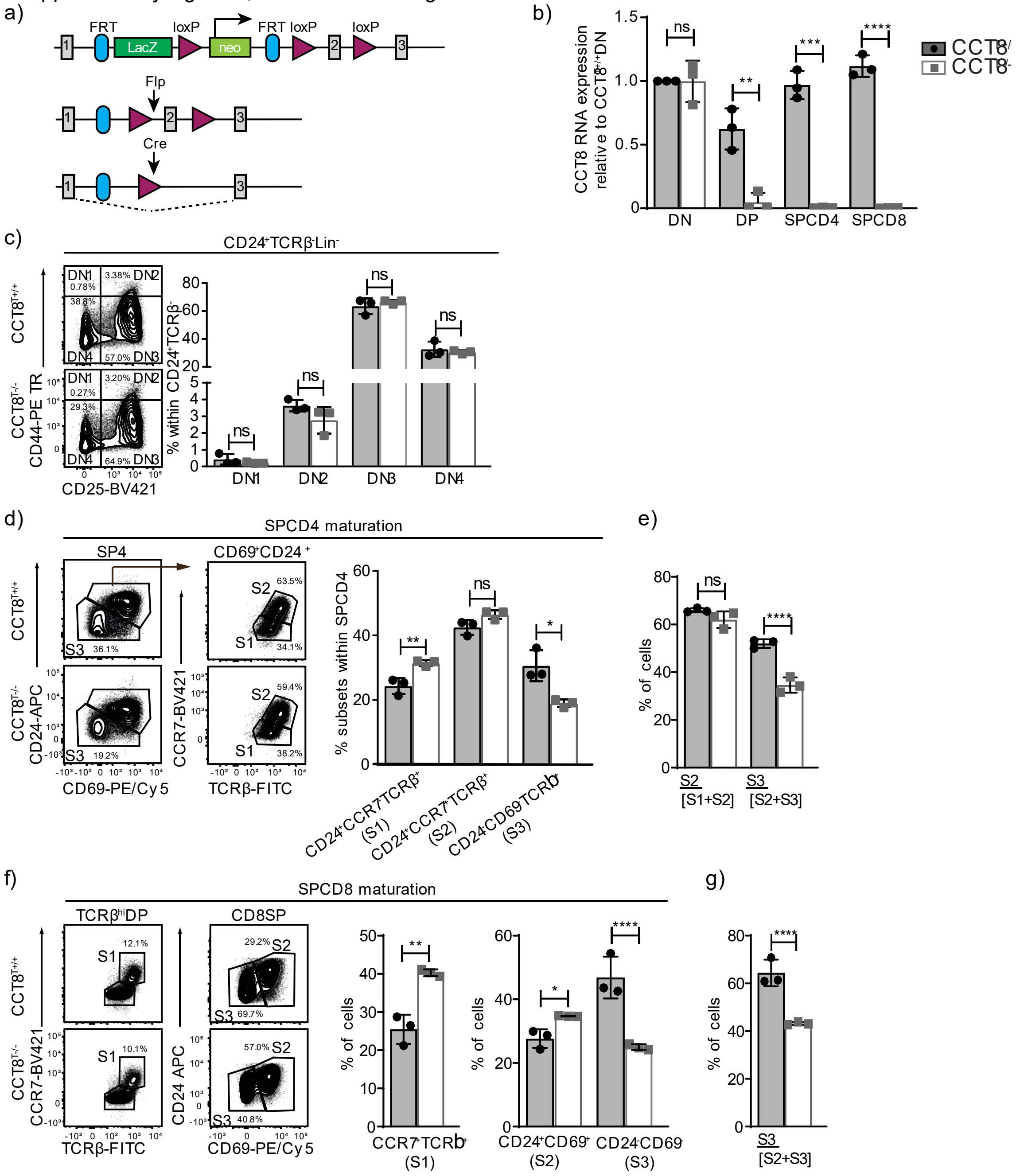

Supplementary Figure 1. Selection and maturation of cortical and medullary thymocytes in the absence of CCT8 expression. Data from CCT8$^{T+/+}$ and CCT8$^{T-/-}$ mice are shown in grey and white bars, respectively. (**a**) Targeting strategy for the conditional deletion of exon 3 of the Cct8 locus. FRT: flippase recognition target; loxP: locus of X-over P1; Flp: Flipase; Cre: Cre-rcompinase. (**b**) Cct8 transcripts in CCT8T+/+ and CCT8T-/- thymocytes at indicated stages of development. qPCR analysis normalised to GPDH and displayed as 2−ΔΔCt CT values. (**c**) Differentiation of linage negative CD24-TCRb- CD4-CD8- thymocytes. Lineage negativity is defined as the absence of CD11b, CD11c, Gr1, CD19, CD49b, F4/80, NK1.1, GL3, and Ter119 expression. Left: contour plots and gating, see also Supplementary information. (**d**) SPCD4 thymocyte maturation. Left: Contour plots showing gating. Individual maturational stages, qualified as immature (CD24+CCR7-TCRb+: S1), semimature (CD24+CCR7+TCRb+: S2) and mature (CD24-CD69- TCRb+: S3). (**e**) Maturational progression between sequential developmental stages. (**f**) SPCD8 thymocyte maturation. Left: Contour plots showing gating.Individual maturational stages, qualified as immature (CCR7+TCRb+: S1), semimature (CD24+CD69+: S2) or mature (CD24-CD69-: S3). (**g**) Maturational progression between sequential developmental stages. *p<0.05, **p<0.01, ***p<0.001, ****p<0.0001. Data in panel b-g were calculated by Student's t-test. Bar graphs show the mean ±SD and are representative of one of 2 independent experiments with 3 samples each.

Supplementary figure 2, related to Main Figure 3

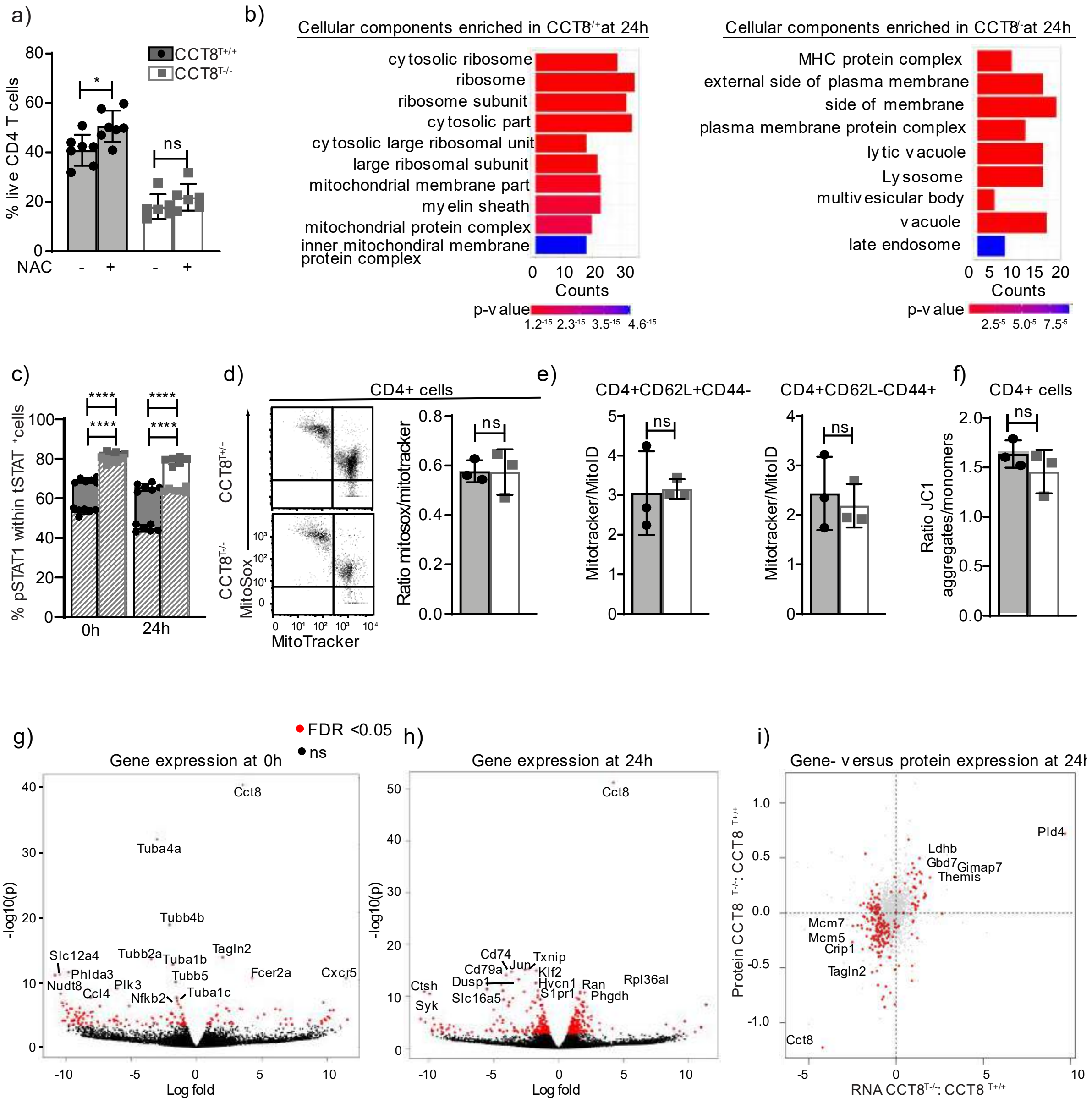

Supplementary Figure 2. Proteomic analysis of activated CD4+ cells.

Bar graphs display data from CCT8$^{T+/+}$ and CCT8$^{T-/-}$ mice in grey and white bars, respectively. (**a**). Percentage of live (i.e. DAPI-) cells after in vitro stimulation for the indicated length of time in the presence or absence of N-acetyl-L-cysteine (NAC: 5mM). (**b**) Go-analysis of differentially expressed genes in CCT8$^{T+/+}$ and CCT8$^{T-/-}$ CD4^{+} T cells stimulated *in vitro* for 24h by CD3 and CD28 crosslinking. (**c**) Frequency of tSTAT1 and pSTAT1 within tSTAT 0hrs and 24hrs upon activation. (**d**) Superoxide mediated oxidation of MitoSOX™. Cells were stimulation for 20h by CD3 and CD28 crosslinking. Representative dot plot analysis (left) of data shown in bar graphs (right). (**e**) Ratio of mitochondrial membrane potential (as assessed by mitoTracker) and mitochondrial mass (as measured by MitoID-red specifically bound to cardiolipin in the inner mitochondrial membrane) in CD62L+CD44- cells and CD62L-CD44+ CD4+ T cells stimulated in vitro for 20h by CD3 and CD28 crosslinking. (**f**) Mitochondrial membrane potential assessed by J-aggregate forming lipophilic cation 5,5′,6,6′-tetrachloro-1,1′,3,3′-tetraethylbenzimidazolcarbocyanine iodide (JC-1) in CD4+ T cells stimulated in vitro for 20h by CD3 and CD28 crosslinking. (**g**) Volcano plots of RNA transcripts in wild type (right) vs. CCT8T-/- left CD4+ T cells (**g**) before (0h) and (ii) after (**h**) in vitro stimulation by CD3 and CD28 crosslinking. (**i**) Correlation of transcriptomic (x-axis) and proteomic (y-axis) ratio data in CD4+ T cells after in vitro stimulation for 24h by CD3 and CD28 crosslinking. Identical transcript/protein entities are shown in red. *p<0.05, **p<0.01, ***p<0.001, ****p<0.0001. Data in panel a was calculated by Student's t test adjusted for multiple comparisons, data in panels c-f were calculated by Student's t test. Bar graphs show the mean ±SD representative of 2 independent experiments with 3 (d-f), 7 (panel a) and 6 (panel c) samples each.

Supplementary Figure 3, related to Main Figure 4

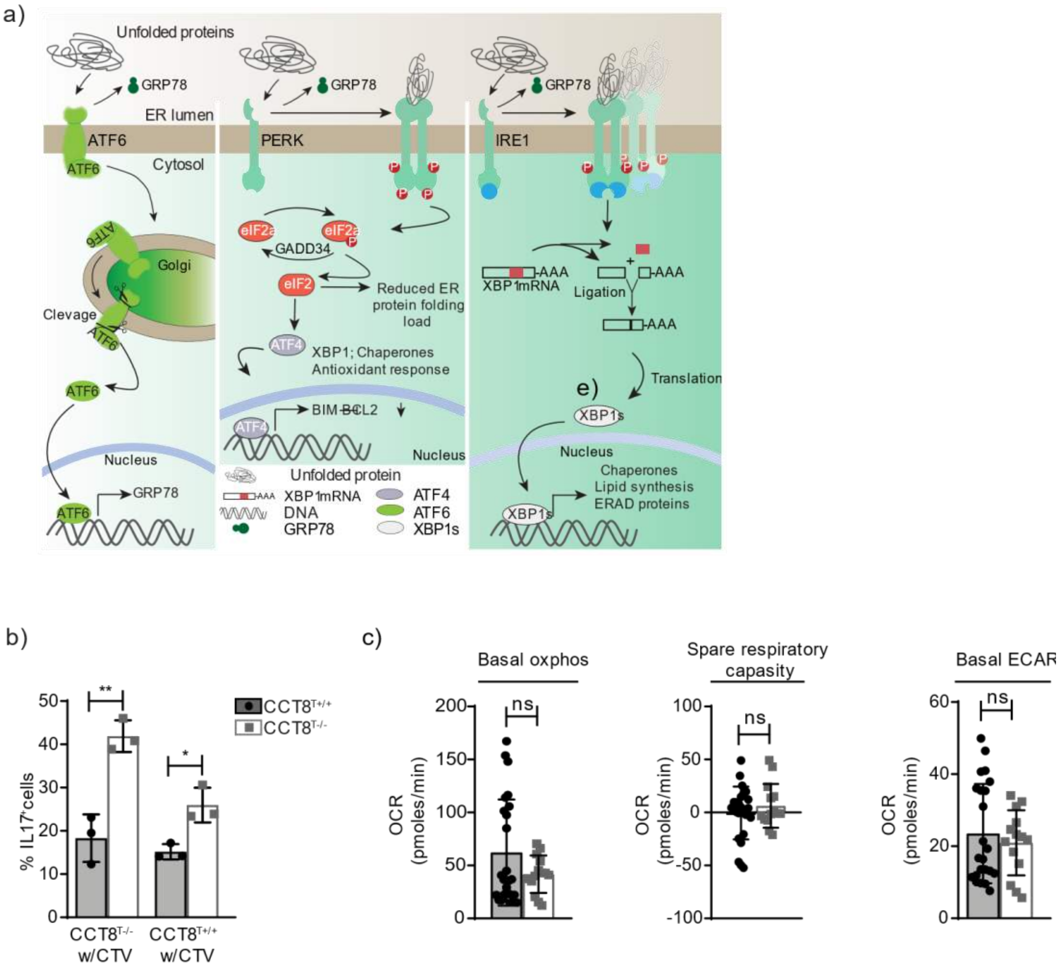

Supplementary Figure 3. Peripheral T cell function in the absence of CCT8 expression.

Schematic of unfolded protein response (UPR) in response to unfolded or misfolded proteins (**a**). Components whose transcripts were examined are shown in bold. (**b**) *In vitro* Th17 polarization of separately labelled CCT8$^{T+/+}$ and CCT8$^{T-/-}$ CD4^{+} T cells stimulated in co-cultures. Left bars show the frequency of Th17 cells in cultures where CCT8$^{T-/-}$ T cells were labelled with cell trace violet (CVT) labelled; right bars show the frequency of Th17 cells in cultures where CCT8$^{T+/+}$ T cells were labelled with CVT. (**c**) Determination by Seahorse XF Analyser of basal oxidative phosphorylation, spare respiratory capacity, and basal extracellular acidification rate (ECAR) in CCT8$^{T+/+}$ and CCT8$^{T-/-}$ CD4^{+} T cells stimulated for 20h by CD3 and CD28 crosslinking. OCR: oxygen consumption rate. *p<0.05, **p<0.01. Data in panel b was calculated by Student's t test, and data in panel c was calculated by Student's t test adjusted for multiple comparisons. Bar graphs show the mean ±SD representative of two independent experiments with three replicates (panel b) and display the combined results of three independent experiments with 14 CCT8$^{T+/+}$ samples and 23 CCT8$^{T-/-}$ samples (panel c).

Supplementary Figure 4, related to Main Figure 5

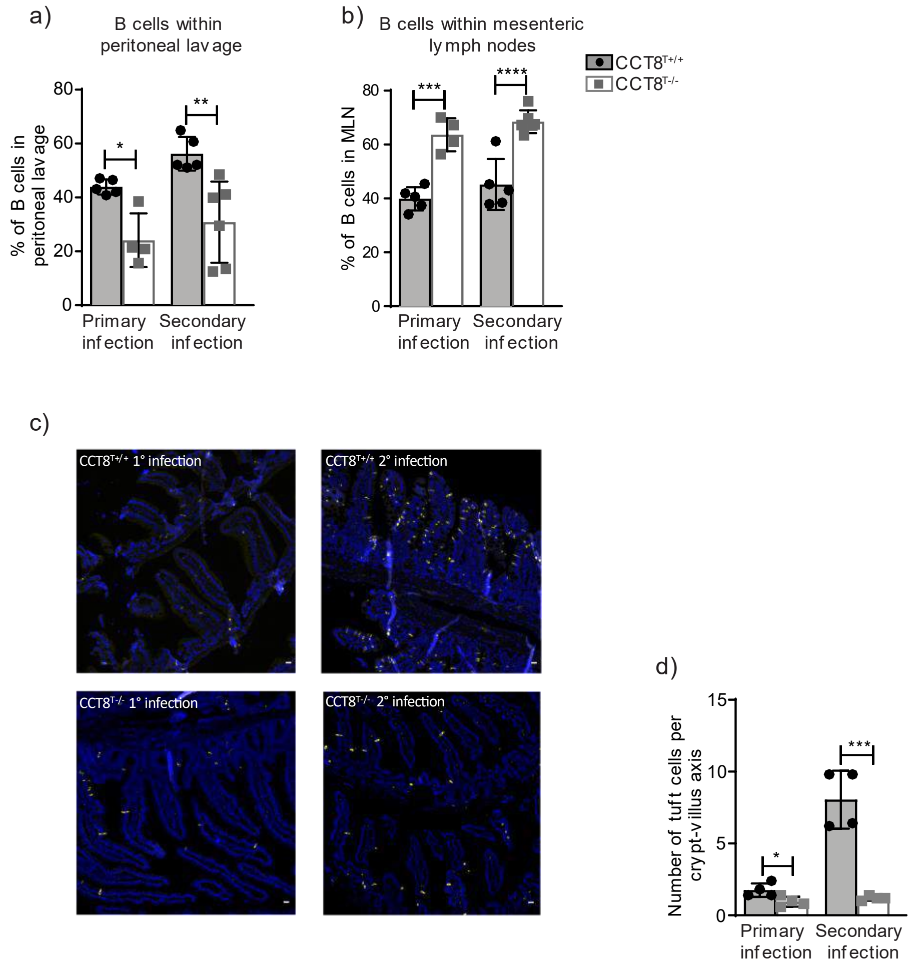

Supplementary Figure 4. The response to *H. polygyrus* infection in CCT8-deficient mice.

Frequency of B cells in peritoneal lavage (**a**) and the mesenteric lymph nodes (**b**) of CCT8$^{T+/+}$ (grey bars) and CCT8$^{T-/-}$ mice (white bars). (**b**) Tuft cells in the small intestine of *H. polygyrus* infected CCT8$^{T+/+}$ and CCT8$^{T-/-}$ mice 14 days after primary and re-infection as indicated. (**c**) Sections shown represent data from mice with median egg counts in each experimental group. For Dclk1 staining: yellow; DAPI: blue. Scale bar in right lower corner represents 20 µm. (**d**) Number of tuft cells in wild type (grey bars) and mutant (white bars) mice. *p<0.05, **p<0.01, ***p<0.001, ****p<0.0001. Data was calculated by Student's t test adjusted for multiple comparison. Bar graphs in panel a show the mean ±SD representative of one of 2 independent experiments with at least 4 samples (CCT8$^{T+/+}$ primary infection: 5 mice, secondary infection 5 mice; CCT8$^{T-/-}$ primary infection 4 mice, secondary infection 6 mice). Data in panel c is representative of 4 separately analysed sections per experimental group.
