# Supplementary material for: The chaperonin CCT8 controls proteostasis essential for T cell maturation, selection, and function
Source: Commun Biol. 2021 Jun 3;4:681. doi: 10.1038/s42003-021-02203-0 (PMC8175432; doi:10.1038/s42003-021-02203-0)
Supplement: Supplementary file 3 — Description of Supplementary Files. [file 42003_2021_2203_MOESM3_ESM.pdf]

Description of Additional Supplementary Files

File name: Supplementary Data 1

Description: Tables containing the data used in the statistical analysis for all figures.

File name: Supplementary Data 2

Description: FACS gating strategy for all panels included in the study.

File name: Supplementary Data 3

Description: Table containing fold difference at timepoint 0hrs and 24hrs in the proteomic and transcriptomic data.

File name: Supplementary Data 4

Description: Key resource table listing antibodies, commercial kits, oligonucleotides, and analysis software used.

File name: Supplementary Data 5

Description: The complete western blot from figure 1a.
