# Supplementary material for: The chaperonin CCT8 controls proteostasis essential for T cell maturation, selection, and function
Source: Commun Biol. 2021 Jun 3;4:681. doi: 10.1038/s42003-021-02203-0 (PMC8175432; doi:10.1038/s42003-021-02203-0)
Supplement: Supplementary file 5 — Supplementary Data 2. [file 42003_2021_2203_MOESM5_ESM.pdf]

Supplemental item 1 relate to Main Figure 1 and Figure S1

a. DN/DP/SPCD4/SPCD8, gating strategy

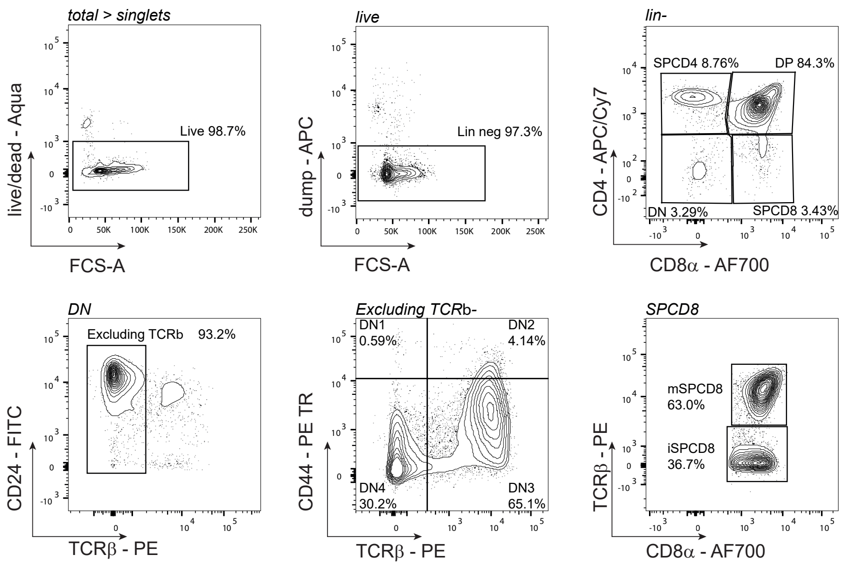

b. positive selection, gating strategy

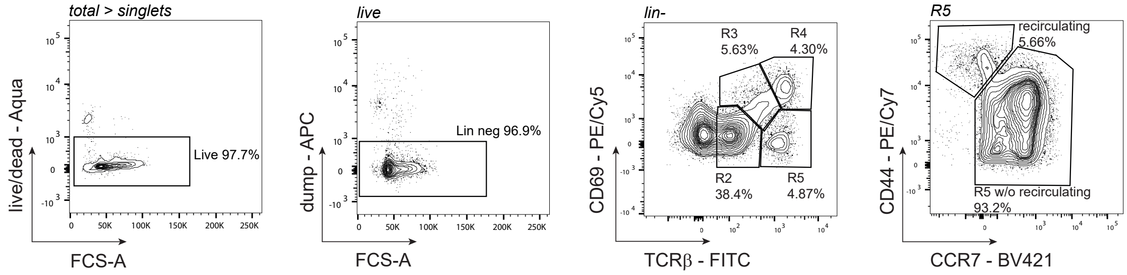

Supplemental item 1a. Representative gating strategy for identification of DN($CD4^-CD8^-$), DP($CD4^+CD8^+$), SPCD4 and SPCD8 by Flow Cytometry.

Supplemental item 1b. Representative gating strategy for identification of stages R2 ($CD69^-TCR\beta^{lo}$), R3 ($CD69^-TCR\beta^{hi}$), R4($CD69^+TCR\beta^{hi}$), R5($CD69^-TCR\beta^{hi}$) by Flow Cytometry.

Supplemental item 2 relate to Main Figure 1 and Figure S1

SM M1 M2 gating strategy

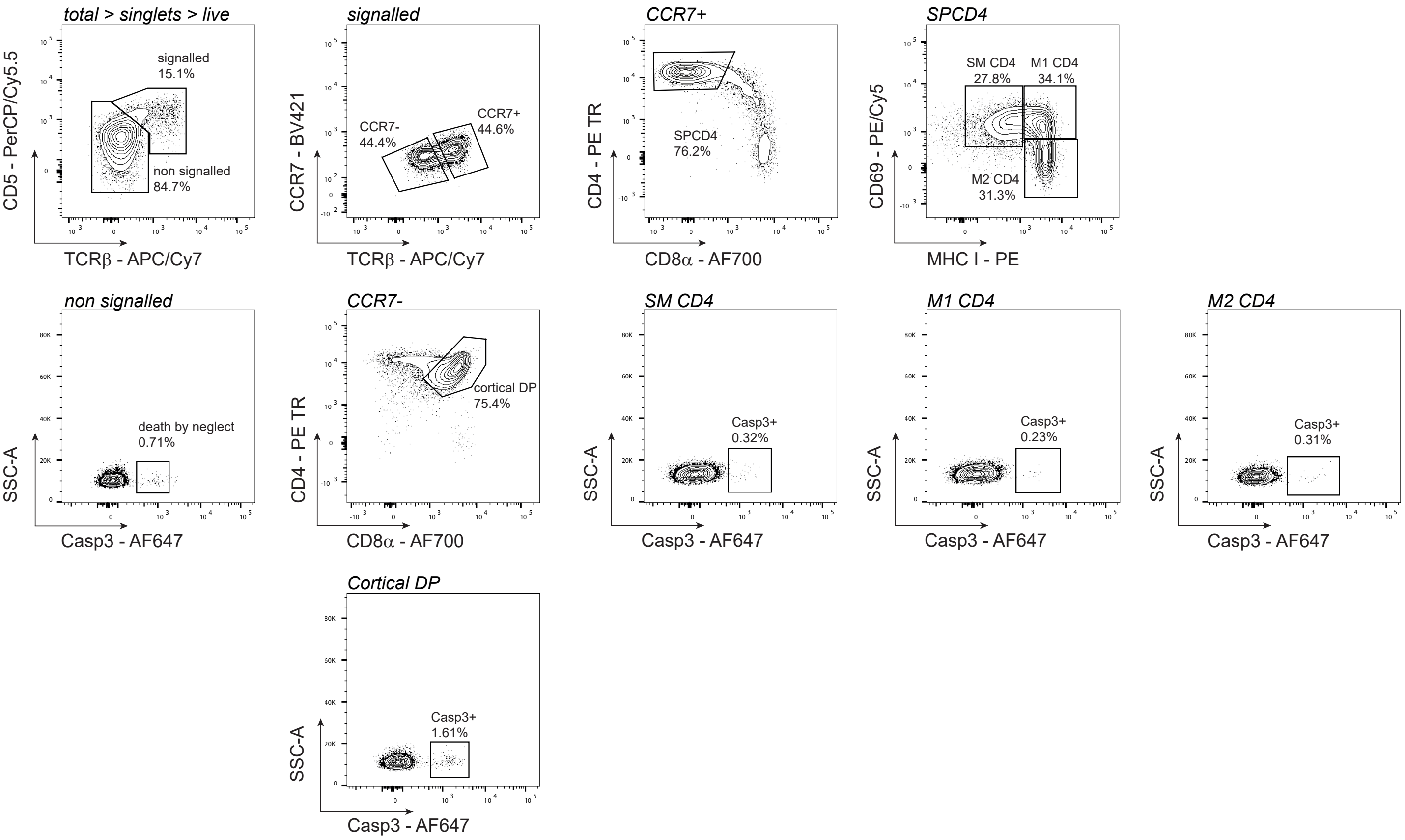

Supplemental item 2. Representative gating strategy for identification of signalled thymocytes (TCRβ^{lo}CD5^{+}), non signalled thymocytes (TCRβ^{hi}CD5^{+}), SM CD4 (TCRβ^{hi}CD5^{+}CCR7^{+}CD69^{+} MHCI^{-}), M1 CD4 (TCRβ^{hi}CD5^{+}CCR7^{+}CD69^{+} MHCI^{+}), M2 CD4 (TCRβ^{hi}CD5^{+}CCR7^{+}CD69^{-} MHCI^{+}) by Flow Cytometry.

Supplemental item 3 relate to Main Figure1 and Figure S1

negative selection, gating strategy

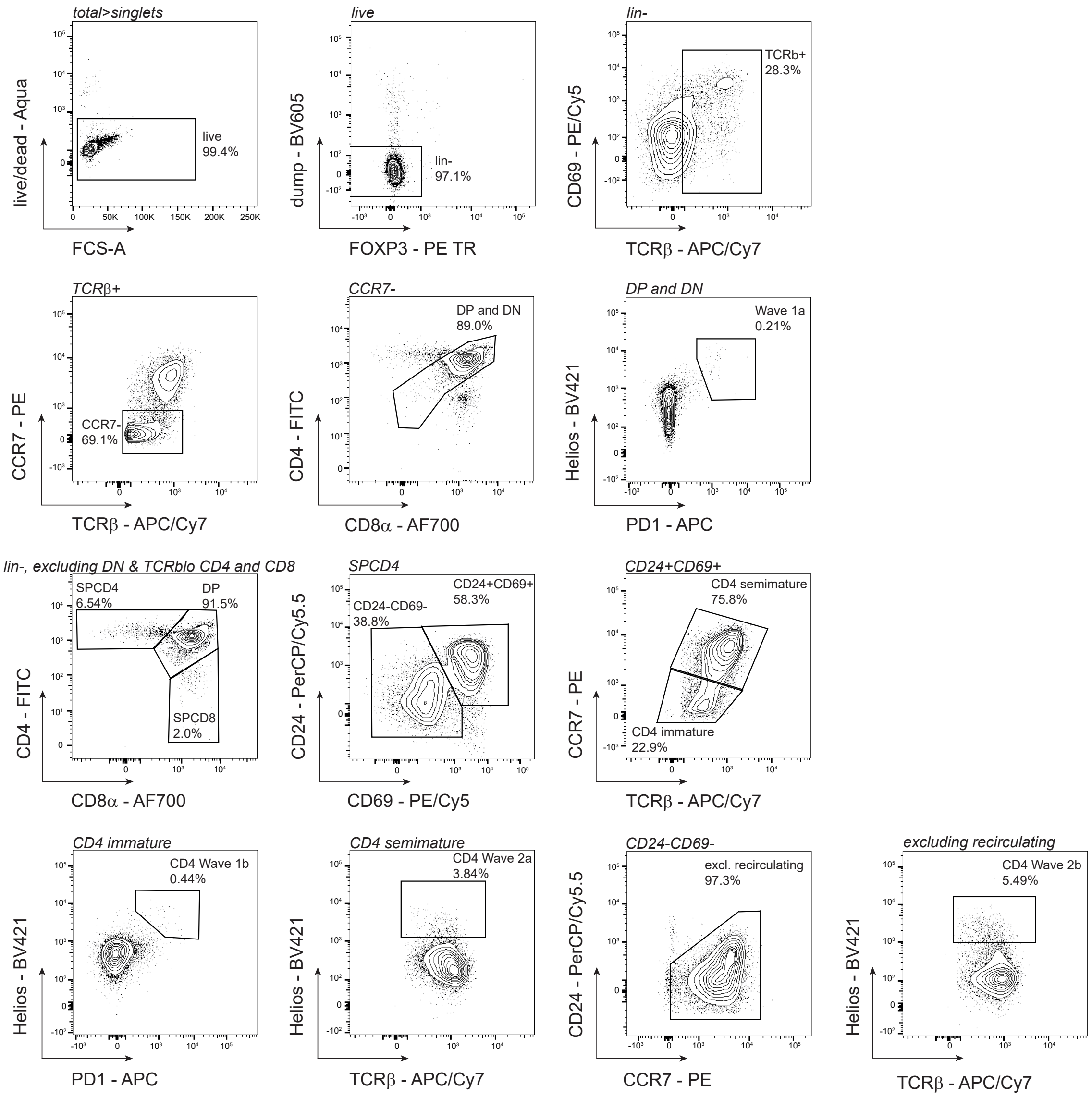

Supplemental item 3. Representative gating strategy for identification of Wave 1a ($TCR\beta^{+}CCR7^{-}CD4^{+/-}CD8^{+/-}Helios^{+}PD1^{+}$), CD4 Wave 1b ($TCR\beta^{+}CCR7^{-}CD4^{+}CD8^{-}CD24^{+}CD69^{+}Helios^{+}PD1^{+}$),CD4 Wave 2a ($TCR\beta^{+}CCR7^{+}CD4^{+}CD8^{-}CD24^{+}CD69^{+}Helios^{+}$), CD4 Wave 2b ($TCR\beta^{+}CCR7^{+}CD4^{+}CD8^{-}CD24^{+}CD69^{+}Helios^{+}$) by Flow Cytometry.

Supplemental item 4, relate to Main Figure 2 and Figure S1

thymic Tregs, gating strategy

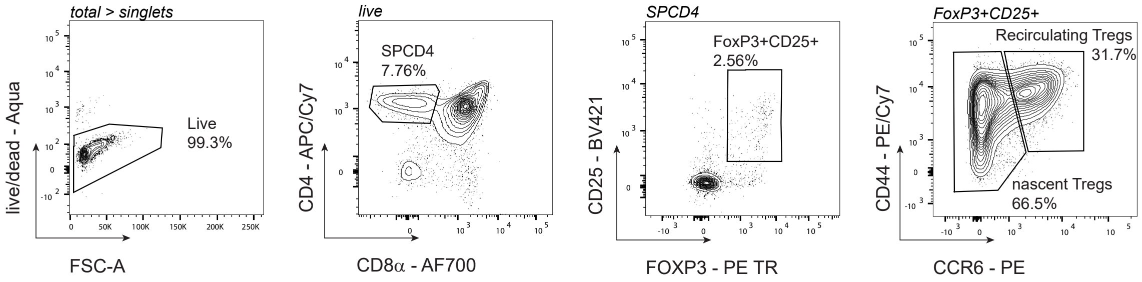

Supplemental item 4. Representative gating strategy for identification of total thymic Tregs ($CD4^{+}CD25^{+}FoxP3^{+}$), nascent Tregs ($CD4^{+}CD25^{+}FoxP3^{+}CCR6^{-}$), recirculating Tregs ($CD4^{+}CD25^{+}FoxP3^{+}CD44^{hi}CCR6^{+}$) by Flow Cytometry.

Supplemental item 5 realte to Main Figure 2

a. memory vs naive and Anergic T cells, gating strategy

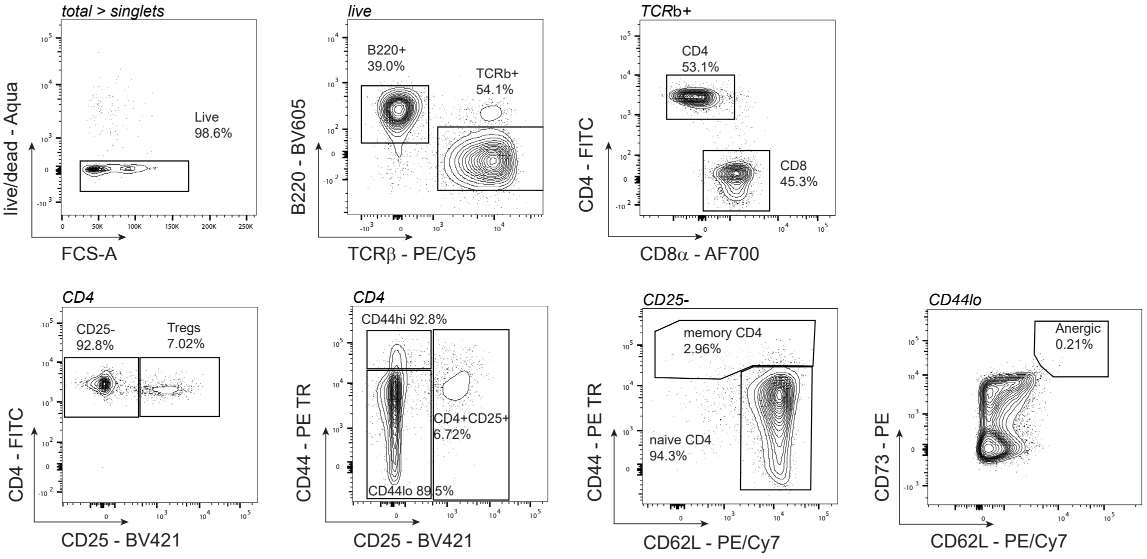

b. peripheral Tregs, gating strategy

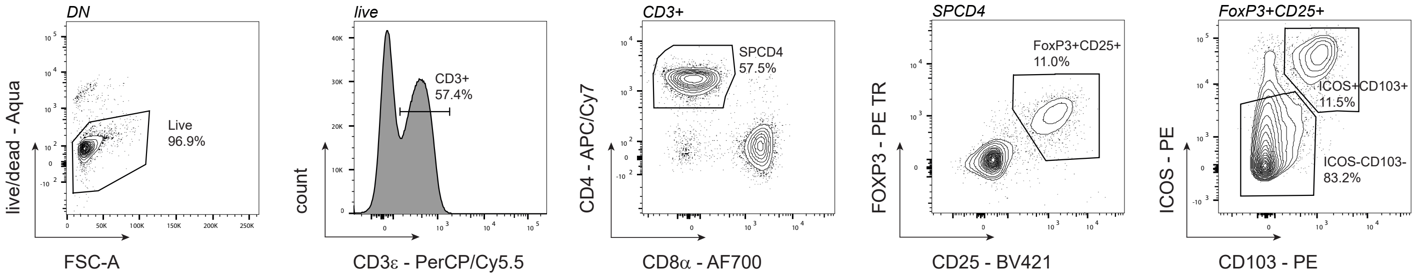

Supplemental item 5a. Representative gating strategy for identification of peripheral B cells ($TCR\beta^{-}B220^{+}$), CD4 ($TCR\beta^{+}CD4^{+}CD8^{-}CD25^{-}$), memory CD4 ($TCR\beta^{+}CD4^{+}CD8^{-}CD25^{-}CD44^{hi}CD62L^{-}$), naive CD4 ($TCR\beta^{+}CD4^{+}CD8^{-}CD25^{-}CD44^{hi}CD62L^{-}$), anergic CD4 ($TCR\beta^{+}CD4^{+}CD44^{lo}FR4^{+}CD73^{+}$) by Flow Cytometry.

Supplemental item 5b. Representative gating strategy for identification of peripheral recirculating regulatory Tregs ($CD3^{+}CD4^{+}CD25^{+}FoxP3^{+}ICOS^{+}CD103^{+}$) by Flow Cytometry.

Supplemental item 6 relate to Main Figure 3 and Figure S2

a. tSTAT1 and pSTAT1, gating strategy

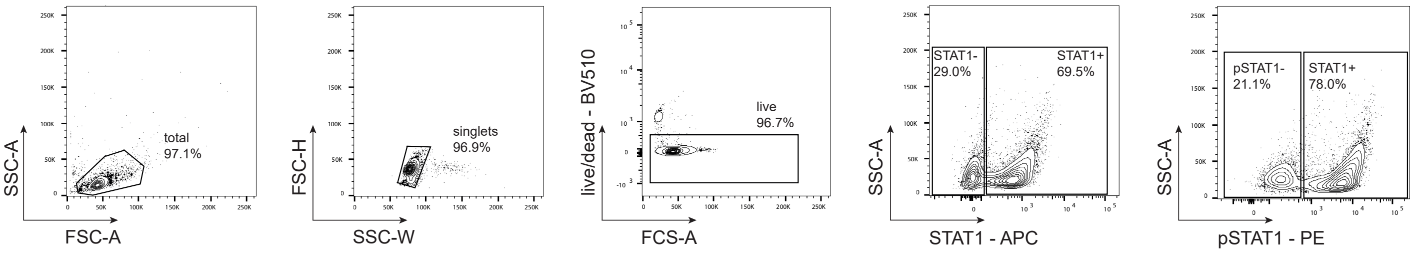

b. MitoSox/MitoTracker, gating strategy

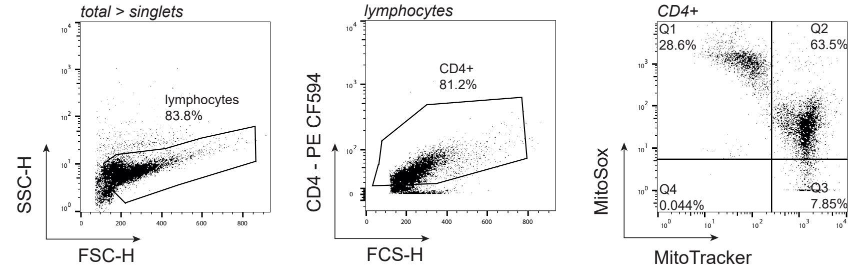

c. JC1, gating strategy

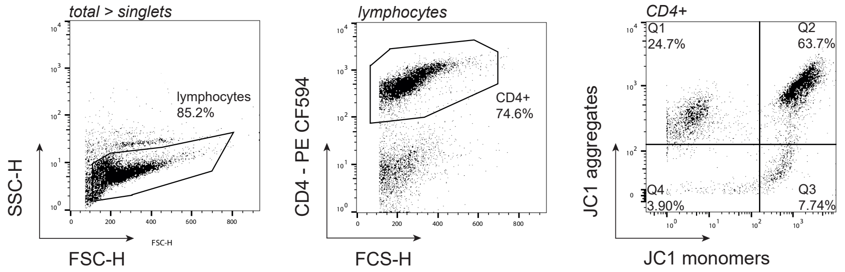

Supplemental item 6a. Representative gating strategy for identification and measurement of total STAT1 and pSTAT1 in naive CD4 cells by Flow Cytometry.

Supplemental item 6b. Representative gating strategy for measurement of superoxide/mithochondial ROS vs. total mitochondria in peripheral naive CD4 cells by Flow Cytometry.

Supplemental item 6c. Representative gating strategy for measurement of JC1 aggregates vs. monomers in peripheral naive CD4 cells by Flow Cytometry.

Supplemental item 7 relate to Main Figure 4

a. Th1 polarisation

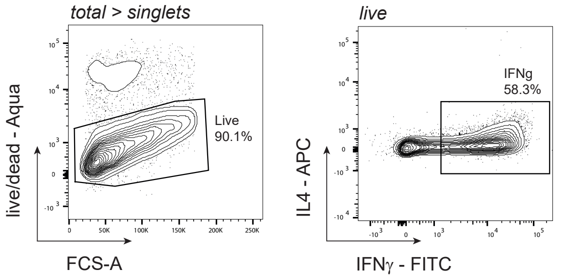

b. Th2 polarisation

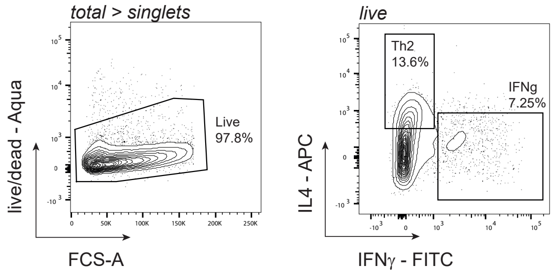

c. Treg polarisation

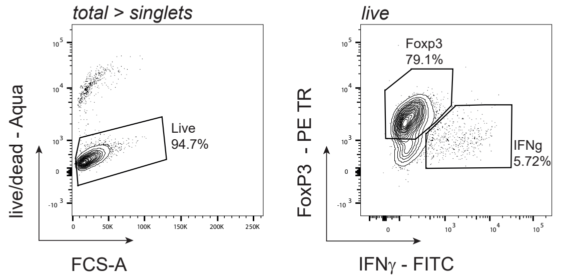

d. Th17 polarisation

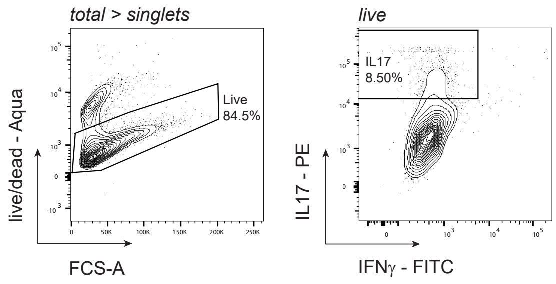

Supplemental item 7. Representative gating strategy for measurement of cytokines productions in a. Th1, b. Th2, c. Treg, d. Th17 polarisation assays in peripheral naive CD4 cells by Flow Cytometry.

Supplemental item 8 relate to Main Figure 5 and Figure S4

a. T cell gating strategy

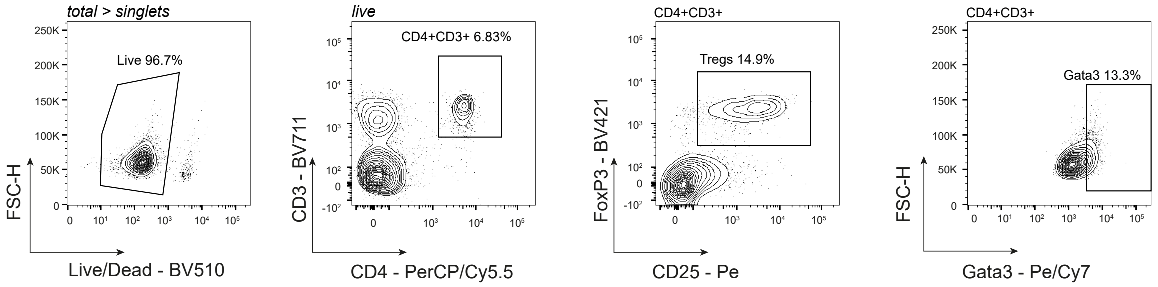

b. Myeloid gating strategy

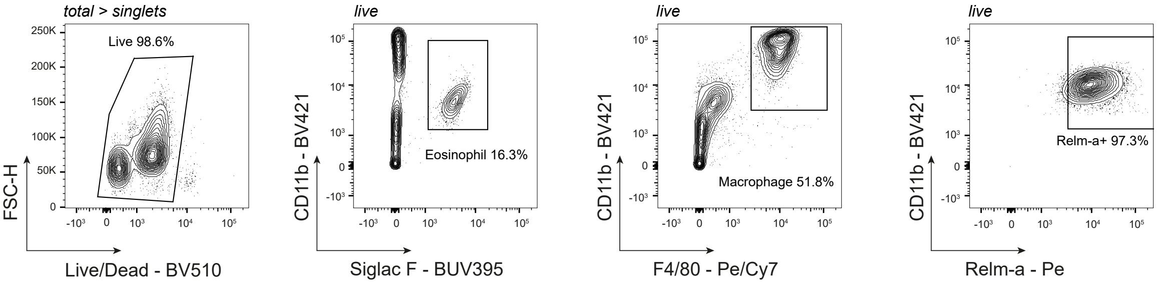

c. B cell gating strategy

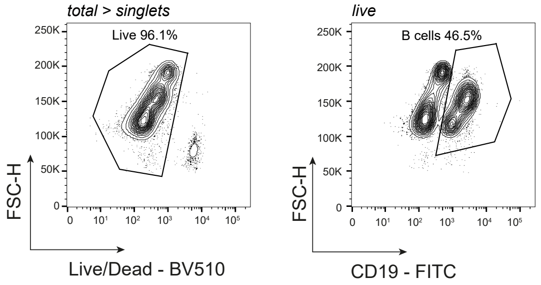

d. ILC2 gating strategy

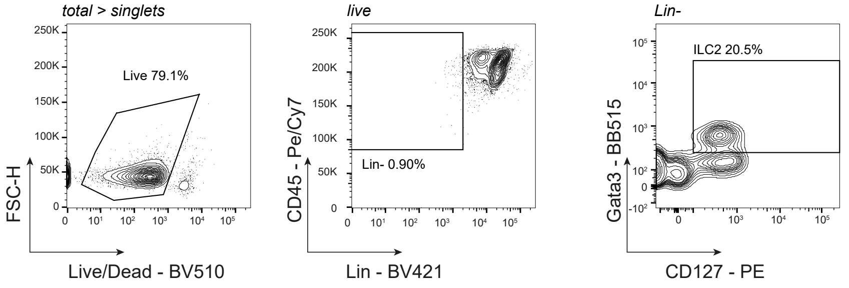

Supplemental item 8a. Representative gating strategy for identification of T cells by Flow Cytometry.

Supplemental item 8b. Representative gating strategy for identification of Myeloid cells by Flow Cytometry.

Supplemental item 8c. Representative gating strategy for identification of B cells by Flow Cytometry.

Supplemental item 8b. Representative gating strategy for identification of ILC2 cells by Flow Cytometry.
