# Supplementary material for: The chaperonin CCT8 controls proteostasis essential for T cell maturation, selection, and function
Source: Commun Biol. 2021 Jun 3;4:681. doi: 10.1038/s42003-021-02203-0 (PMC8175432; doi:10.1038/s42003-021-02203-0)
Supplement: Supplementary file 8 — Supplementary Data 5. [file 42003_2021_2203_MOESM8_ESM.pdf]

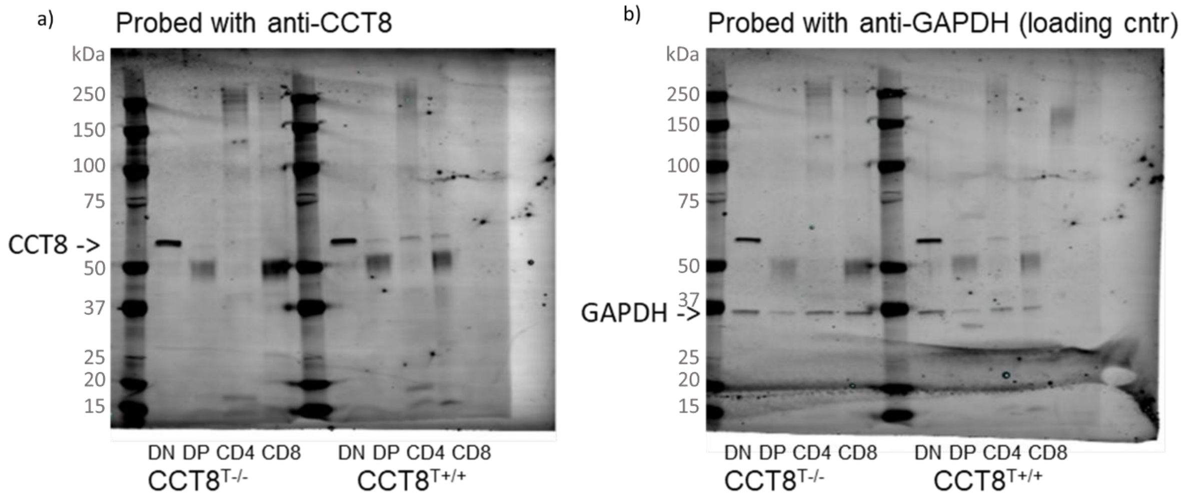

The full Western blot relating to Figure 1a. the Western blot analyses which form the basis of Figure 1a showing CCT8 (molecular weight 59 kDa) (a) and the loading control GAPDH (molecular weight 37 kDa) within the thymic populations DN, DP, SPCD4 (CD4) and SPCD8 (CD8) (b). Please note that the membrane was probed first with an anti-CCT8 antibody (a) and then with an anti-GAPDH antibody (b).
