# Supplementary material for: The chaperonin CCT8 controls proteostasis essential for T cell maturation, selection, and function
Source: Commun Biol. 2021 Jun 3;4:681. doi: 10.1038/s42003-021-02203-0 (PMC8175432; doi:10.1038/s42003-021-02203-0)
Supplement: Supplementary file 9 — Reporting summary. [file 42003_2021_2203_MOESM9_ESM.pdf]

nature research

Corresponding author(s): Georg A. Holländer

Last updated by author(s): Apr 16, 2021

Reporting Summary

Nature Research wishes to improve the reproducibility of the work that we publish. This form provides structure for consistency and transparency in reporting. For further information on Nature Research policies, see our Editorial Policies and the Editorial Policy Checklist.

Statistics

For all statistical analyses, confirm that the following items are present in the figure legend, table legend, main text, or Methods section.

n/a	Confirmed	
☐	☒	The exact sample size (n) for each experimental group/condition, given as a discrete number and unit of measurement
☐	☒	A statement on whether measurements were taken from distinct samples or whether the same sample was measured repeatedly
☐	☒	The statistical test(s) used AND whether they are one- or two-sided<br>*Only common tests should be described solely by name; describe more complex techniques in the Methods section.*
☒	☐	A description of all covariates tested
☐	☒	A description of any assumptions or corrections, such as tests of normality and adjustment for multiple comparisons
☐	☒	A full description of the statistical parameters including central tendency (e.g. means) or other basic estimates (e.g. regression coefficient) AND variation (e.g. standard deviation) or associated estimates of uncertainty (e.g. confidence intervals)
☒	☐	For null hypothesis testing, the test statistic (e.g. F, t, r) with confidence intervals, effect sizes, degrees of freedom and P value noted<br>*Give P values as exact values whenever suitable.*
☒	☐	For Bayesian analysis, information on the choice of priors and Markov chain Monte Carlo settings
☒	☐	For hierarchical and complex designs, identification of the appropriate level for tests and full reporting of outcomes
☒	☐	Estimates of effect sizes (e.g. Cohen's d, Pearson's r), indicating how they were calculated

Our web collection on statistics for biologists contains articles on many of the points above.

Software and code

Policy information about availability of computer code

Data collection	FACS diva, INSPIRE 200 software (Amnis Corporation), Leica LAS AF.
Data analysis	Flowo v10 (Analysis of flow data), Odyssey imaging system (Licor) for western blot analysis, image processing software Fiji (microscopy data), MaxQuant1.6.2.6 (proteomic analysis), edgeR (transcriptomic analysis), Cluster Profiler (transcriptomic analysis), Wave desktop software (Seahorse data analysis), IDEAS v 6.2 software (image stream analysis).

For manuscripts utilizing custom algorithms or software that are central to the research but not yet described in published literature, software must be made available to editors and reviewers. We strongly encourage code deposition in a community repository (e.g. GitHub). See the Nature Research guidelines for submitting code & software for further information.

Data

Policy information about availability of data

All manuscripts must include a data availability statement. This statement should provide the following information, where applicable:

- Accession codes, unique identifiers, or web links for publicly available datasets
- A list of figures that have associated raw data
- A description of any restrictions on data availability

RNAseq data are available at the NCBI Gene Expression Omnibus under accession number GEO: GSE147209.
The mass spectrometry proteomics data have been deposited to the ProteomeXchange Consortium via the PRIDE partner repository with the dataset identifier PXD018834, Reviewer account details:
Username: reviewer51034@ebi.ac.uk
Password: FBZnFVxe
All figures have associated raw data

Further information and requests for resources and reagents should be directed to and will be fulfilled by the Lead Contact, Georg Holländer (georg.hollander@paediatrics.ox.ac.uk).

Field-specific reporting

Please select the one below that is the best fit for your research. If you are not sure, read the appropriate sections before making your selection.

- [x] Life sciences
- [ ] Behavioural & social sciences
- [ ] Ecological, evolutionary & environmental sciences

For a reference copy of the document with all sections, see nature.com/documents/nr-reporting-summary-flat.pdf

Life sciences study design

All studies must disclose on these points even when the disclosure is negative.

Sample size	All experiments have been carried out at least two times with biological triplicates in each group of the independent experiments.
Data exclusions	No data was excluded from analysis.
Replication	All experiments were reproduced successfully and all findings were reproducible. The number of individual, independent experiments are indicated in the figure legends.
Randomization	The experimental groups were determined by different genotype (i.e. wild type versus homozygous CCT8-deficient) and were matched for general genetic background, age and gender. Control mice were non-Cre lox::lox animals, hence allowing the analysis of litter mates of individual matings with one parent heterozygous for the expression of Cre.
Blinding	The two experimental groups (i.e. wild type versus homozygous CCT8-deficient) provided an obvious phenotype upon autopsy for the phenotypic analyses that blinding was not possible. In the infection experiments, blinding was not achieved due to the large worm burden in the CCT8-deficient mice infected.

Reporting for specific materials, systems and methods

We require information from authors about some types of materials, experimental systems and methods used in many studies. Here, indicate whether each material, system or method listed is relevant to your study. If you are not sure if a list item applies to your research, read the appropriate section before selecting a response.

Materials & experimental systems

n/a	Involved in the study	
	x	Antibodies
x		Eukaryotic cell lines
x		Palaeontology and archaeology
	x	Animals and other organisms
x		Human research participants
x		Clinical data
x		Dual use research of concern

Methods

n/a	Involved in the study	
x		ChIP-seq
	x	Flow cytometry
x		MRI-based neuroimaging

Antibodies

Antibodies used	Please see attached list of antibodies used in the study.
Validation	All antibodies used in the study have been previously validated and titrated in the lab, and are extensively used in the lab for various projects.

Animals and other organisms

Policy information about studies involving animals; ARRIVE guidelines recommended for reporting animal research

Laboratory animals	The Cct8 KO mouse model (Cct8tm1a(KOMP) Wtsi, Project ID CSD45380i) on a C57/Bl6 background was obtained from the KOMP Repository (www.komp.org) and rederived by the Wellcome Trust Sanger Institute (WTSI). Females and males were used at 4-10 weeks of age.
Wild animals	The study does not involve wild animals.
Field-collected samples	The study does not involve field-collected samples.

Ethics oversight	Animal experiments were performed according to institutional and UK Home Office regulations.

Note that full information on the approval of the study protocol must also be provided in the manuscript.

Flow Cytometry

Plots

Confirm that:

- [x] The axis labels state the marker and fluorochrome used (e.g. CD4-FITC).
- [x] The axis scales are clearly visible. Include numbers along axes only for bottom left plot of group (a 'group' is an analysis of identical markers).
- [x] All plots are contour plots with outliers or pseudocolor plots.
- [x] A numerical value for number of cells or percentage (with statistics) is provided.

Methodology

Sample preparation	Thymus, spleen and lymph nodes from mice were harvested and the immune cells were brought into single cell suspension by gentle dissociation of the tissue using nylon filter mesh.
Instrument	BD FACS Aria III
Software	BD FACS Diva used for sorted populations, Three star Flow Jo version 10 used for analysis
Cell population abundance	The abundance of the relevant cell population after sorting was over 96%. This was determined by running a fraction of the sorted population on the FACS again after sorting and/or magnetic selection.
Gating strategy	Please see the gating strategy file provided with the manuscript.

- [x] Tick this box to confirm that a figure exemplifying the gating strategy is provided in the Supplementary Information.
